# Reliability and Validity of the Utrecht Tasks for Attention in Toddlers Using Eye Tracking (UTATE)

**DOI:** 10.3389/fpsyg.2020.01179

**Published:** 2020-06-08

**Authors:** Anneloes L. van Baar, Marjanneke de Jong, Martine Maat, Ignace T. C. Hooge, Lilly Bogičević, Marjolein Verhoeven

**Affiliations:** ^1^Department of Child and Adolescent Studies, Utrecht University, Utrecht, Netherlands; ^2^Experimental Psychology, Helmholtz Institute, Utrecht University, Utrecht, Netherlands

**Keywords:** reliability, validity, attention, eye tracking, toddlers, UTATE

## Abstract

Attention problems hinder many children in their cognitive and social emotional development. Children at risk for developmental problems, like preterm born infants, are specifically known for attention difficulties. Early identification of attention difficulties is important for application of appropriate stimulation in trying to reduce further problems. Specifically designed instruments with good psychometric characteristics are needed to show difficulties in attention, that may contribute to early identification. The Utrecht Tasks of Attention in Toddlers using Eye tracking (UTATE) is an instrument to measure orienting, alerting and executive attention capacities in young children. Reliability and validity of the UTATE are specifically addressed in three studies, reported in this paper. A sample of 95 term born children assessed at 18 months of age was used that provided data for both the second and third study reported here. In addition, three other small samples were used, of which the first consisted of 12 children at 18 months with test-retest data available that are reported in the first study. Two other samples that were used in the third study, consisted of 14 children measured at 12 months, and 15 children examined at 24 months. The UTATE resulted in reliable information on eye movements and some first support for construct and predictive validity was found. Low scores on the UTATE at 18 months were found to be related to slower cognitive development as measured with the Bayley-III-NL at 24 months. Furthermore, a first indication that the UTATE is able to detect some age differences in attention was found. It is concluded that the UTATE can be used to study attention capacities in toddlers that underlie cognitive functioning and development, but further research is necessary.

## Introduction

Many children experience problems in attention development (e.g., Mahone and Schneider, 2012). While development of attention capacities starts already early in life, problems are usually not recognized before school entry (Ruff and Rothbart, 1996; Atkinson and Braddick, 2012). To be able to detect attention problems at an earlier age, reliable and valid measures are needed, that objectively measure attention capacities. For this reason, the Utrecht Tasks for Attention in Toddlers using Eye tracking (UTATE) was developed (De Jong et al., 2016b). The UTATE consists of four tasks that are administered on an eye tracker and intends to measure functioning of three theoretically distinguished attention systems: orienting, alerting, and executive attention (Posner and Petersen, 1990). *Orienting* concerns the ability to activate attention and shift between visual targets, as becomes evident by relocating the gaze (Posner and Petersen, 1990; Atkinson and Braddick, 2012). *Alerting* is a skill that consists of the ability to attain and sustain attention for important cues in the environment. *Executive attention* is considered to be a more internal and endogenous system of attention, which entails directed attention and inhibition of behavior (Colombo, 2001; Atkinson and Braddick, 2012).

Several studies provided information regarding reliability and validity of the UTATE. In a pilot study was shown that the UTATE was feasible for use with 18-month-old children: the toddlers cooperated well during the procedure, and the data was of good quality and measured individual variation (De Jong et al., 2016b). Furthermore, sufficient split half reliability was found (De Jong et al., 2015, 2016b). In a second study, factorial validity of the UTATE was shown by a confirmatory factor analyses providing evidence for three underlying factors (i.e., orienting, alerting, and executive attention), as was expected based on the theory underlying the design of the tasks (De Jong et al., 2016a). Another study showed first evidence for clinical validity as the UTATE differentiated between a group of children at risk for attention difficulties (i.e., preterm children) and a typically developing group of children (De Jong et al., 2015). Further evaluation of the potential and the psychometric characteristics of the UTATE is important, also to allow other researchers to use our information regarding studies with eye tracking to evaluate attention capacities in toddlers and perhaps even develop improved instruments based on the UTATE. Therefore, the test-retest reliability (the focus of study 1) as well as the convergent, divergent and predictive validity (the focus of study 2) and an exploration of the results at different ages (the focus of study 3) of the UTATE are reported in the current paper.

Reliability indicates the consistency of an instrument, which can be measured in different ways, like split half reliability and test-retest reproducibility. Split-half reliability is a measure of the internal consistency of an instrument. For the UTATE, split-half reliability was studied by deriving the outcome variables separately for the even and odd numbered trials of the tasks. The correlation between the variables of the even and odd numbered trials indicated the strength of reliability (Field, 2009). Although this method already gave a first impression of reliability of the UTATE for toddlers at the age of 18 months, a drawback was that due to splitting the data, only half of the data is used to compute split-half reliability. In addition, for one of the tasks (i.e., delayed response task), making an appropriate split was not possible due to one of the outcome variables that could not be evenly divided among even and odd numbered trials. Therefore, another type of reliability of the UTATE will be investigated in this paper, again with toddlers at the age of 18 months, in study 1: test-retest reliability. Test-retest reliability is a measure of consistency and is examined by administering an instrument twice within a short time span, like 2 weeks, which is especially important for a construct as assessed by the UTATE, as attention skills in early infancy and toddlerhood are subjected to developmental and maturational changes. Strong correlations between measurements at two moments within a short period of time, is seen as proof of test-retest reliability.

Construct validity refers to the ability of an instrument to actually measure a certain construct (Cohen and Swerdlik, 2010), in this case attention. To determine the construct validity, both convergent and divergent validity have to be found and these will be addressed in study 2. Convergent validity indicates that a measure is equally suitable in identifying attention skills as other measures of attention (Cohen and Swerdlik, 2010). To investigate convergent validity, the *orienting* system as measured by the UTATE will be compared with mother-reported attention shifting skills of toddlers. The *alerting* system as measured by the UTATE will be compared to mother-reported attention focusing skills, and to observed on-task persistence of the toddlers during a free and structured play setting, coded by trained professionals. The *executive attention* system as measured by the UTATE is compared to mother-reported effortful control: a temperament dimension suggested to be closely related to executive attention (Rothbart et al., 2007). A moderately sized correlation between the UTATE and other measures of attention, evaluated with different kinds of instruments, is seen as proof of convergent validity. Divergent validity is accepted when the attention systems as measured by the UTATE are not, or less strongly related to constructs not supposed to reflect attention (Cohen and Swerdlik, 2010). As attention capacities underlie many cognitive activities, it is difficult to determine constructs to which it might not be related (Atkinson and Braddick, 2012). The orienting, alerting and executive attention systems of the UTATE will be compared to mother-reported social-emotional functioning and communication skills, with which no, or only weak relationships are expected.

Predictive validity of the UTATE will be found when the attention systems are related to measures of attention capacities and developmental outcome based on attention capacities, like cognitive capacities at older ages. For all three attention systems, predictive validity is studied by comparing the UTATE measure at 18 months of age to cognitive functioning assessed with a developmental test at 24 months of age in study 2. Next to that, orienting, alerting, and executive attention were compared to respectively mother-reported attention shifting, attention focusing, and effortful control measured at 24 months of age.

Part of validity of an instrument is also whether it is able to detect expected developmentally specific patterns. Although there are no studies that empirically tested the development of orienting, alerting, and executive attention during the second year of life, theoretically it is expected that attention capacities change and improve during the first years of life (e.g., Ruff and Rothbart, 1996). In study 3, we explore whether the UTATE is feasible for use with 12- and 24-month-old children. In addition is studied if the UTATE is capable of detecting age differences in attention skills by comparing the performance of 12-, 18-, and 24-month-old children. In this way a first impression of the potential of the UTATE in studying age related development of attention capacities is presented.

## Study 1 – Reliability

### Research Question

To what extent is the performance on the UTATE related to performance on the UTATE within the following 2 weeks? In other words, is the test-retest reliability of the UTATE adequate?

### Materials and Methods

#### Participants and Procedure

The participants for this study formed a convenience sample and they were acquired by students in their own network, who asked parents with children aged around 18 months to participate in this study consisting of two assessments with the UTATE and answering a short questionnaire on demographic background characteristics. Parents and caretakers considered the children to be healthy at the time of assessment. One of the children could only be assessed once, because of an unexpected holiday at the second appointment. The sample with data for both measurements consisted of 12 healthy Dutch children aged 16–22 months (*M* = 19.00, *SD* = 1.86, 41.7% boys). The UTATE was administered twice with, on average, 8 days in between (*M* = 8.33 days, *SD* = 2.93, range 6–15). The UTATE was administered in a lab setting (*n* = 1), at their child care centre (*n* = 8), or at home (*n* = 3). For all children, the location was the same at the first and second measurement moment.

The research project was approved by the Medical Ethical committee of the University Medical Centre Utrecht. All parents gave informed consent for their child’s participation.

#### Measures

##### UTATE

The UTATE consists of four tasks: (1) In the *disengagement task*, a stimulus was first presented at the center of the screen, and after 2 s a second stimulus appeared at the left or the right side of the central stimulus. This task included 20 trials. (2) In the *face task*, first two pictures of identical child faces were shown, and after 8.5 s one of the pictures changed into a new picture and stayed on the screen together with the previously shown picture for 8 s. This task consisted of eight trials. (3) In the *alerting task*, a stimulus was presented on the screen for 32 trails and in half the trails, this was preceded by a signaling sound. (4) In the *delayed response task*, a dog was hiding in one out of two doghouses and the child was asked to search for the dog. This is the only task that makes use of an instruction for the child. A voice-over directs the child toward a dog on the screen and tells the child the dog wants to play “hide-and-seek.” The child is told to pay attention, because the dog is going to hide himself. The dog moves to one of two doghouses for 1000 ms, before he disappears. A worm pops up in the center of the screen, accompanied by a little music, to distract the child from the dog houses, and after a delay the child is asked to search for the dog by a voice-over. This task consisted of 18 trials, in which the delay increased with 2 s after three trials, from 0 to 10 s. Details regarding the instrument, apparatus and procedure are described in De Jong et al. (2016b) and in the manual (see Supplementary Material). Fixations were classified with the method described in Hooge and Camps (2013). Thirteen variables were derived from these tasks (see Tables 1, 2). The whole procedure to do the UTATE took about 18 min. Please, also see our manual concerning the procedures we used in the Supplementary Material.

**TABLE 1:** Descriptions of the variables from the four eye-tracker tasks.

Outcome measure	Task	Description
**Orienting system**		
Mean dwell time	DIS, FACE,	Average length of the dwells. A dwell is the length of “one visit in an area of interest [AOI], from entry to exit” (Holmqvist et al., 2011)
Transition rate	DIS, FACE	The number of transitions (i.e., “movement from one AOI to another,” Holmqvist et al., 2011) divided by the total dwell time
Proportion of correct refixations	DIS	A correct refixation indicates that the participant refixated from the central stimulus to the new stimulus after the new stimulus is presented. The proportion of correct refixations is the number of correct refixations divided by the total number of trials in which the child looked at the central stimulus when the new stimulus appeared
Latency	DIS	The average time between appearance of the new stimulus and fixation on the new stimulus in trials in which the participant correctly refixated
**Alerting system**		
Total dwell time	DIS, FACE, AL, DR	Sum of the length of all dwells. A dwell is the length of “one visit in an area of interest [AOI], from entry to exit” (Holmqvist et al., 2011)
Latency difference	AL	Difference between latencies in the trials in which a signal preceded the appearance of the stimulus (i.e., signal trials) and the trials in which the stimulus appeared without signal (no-signal trials)
**Executive attention system**		
Correct searches	DR	The number of trials in which the child looked at the correct dog house directly in response to the voice over asking where to find the dog
Mean delay	DR	The mean delay between hiding and the instruction to seek the dog in the trials in which the child correctly searched for the dog

*DIS, disengagement task; FACE, face task; AL, alerting task; DR, delayed response task.*

**TABLE 2:** Means and standard deviations at the first and second measurement moment and test-retest reliability: correlations between the variables measured at the first and second moment.

	First moment Mean (*SD*)	Second moment Mean (*SD*)	Pearson correlation	Power
**Orienting**				
1. DIS mean dwell	1409 (239)	1558 (436)	0.55*	0.683
2. DIS latency	622 (211)	617 (212)	0.46	0.510
3. DIS proportion correct	0.93 (0.14)	0.95 (0.07)	0.55*	0.683
4. DIS transition rate	0.48 (0.15)	0.48 (0.24)	0.85*	0.998
5. FACE mean dwell	1286 (265)	1194 (195)	0.49	0.566
6. FACE transition rate	0.61 (0.11)	0.60 (0.20)	–0.07	0.078
**Alerting**				
7. DIS total dwell	80,067(18,615)	86,280(33,379)	0.49	0.566
8. FACE total dwell	68,288(24,343)	75,587(33,133)	0.53*	0.644
9. AL total dwell	47,930(27,199)	46,479(29,309)	0.79*	0.993
10. AL latency difference	−112(680)	322 (601)	0.21	0.171
11. DR total dwell	89,631(20,047)	71,191(29,019)	0.86*	0.999
**Executive attention**				
12. DR correct searches	9.08 (2.78)	8.41 (3.37)	0.71*	0.945
13. DR mean delay	5.63 (0.76)	5.26 (1.62)	0.63*	0.833

DIS, disengagement task; FACE, face task; AL, alerting task; DR, delayed response task. *p < 0.05, one tailed.

#### Statistical Analyses

As test-retest reliability cannot be computed for latent constructs which have to be derived for each sample separately, Pearson’s correlations were computed between the 13 variables that were derived from the UTATE at the first and second measurement moment. The 13 variables are ordered by the latent constructs on which they load (De Jong et al., 2016a). We adopt the criteria used in previous studies including neurocognitive tasks, where correlations between 0.50 and 0.70 were interpreted as “adequate reliability” and above 0.70 as “good reliability” (Kindlon et al., 1995; Kuntsi et al., 2001; Karalunas et al., 2016). SPSS version 25.0 was used for the analysis with α set at 0.05, one tailed, in view of the expected positive correlations. *Post-hoc* power analyses were done using the G^∗^Power tool, version 3.1.9.7 (Faul et al., 2009).

### Results

The means, standard deviations and correlations between the variables at both measurement moments as well as the power of the results are presented in Table 2 for each attention system.

#### Orienting

For the variables that measure functioning of the orienting system, test-retest reliability was good for transition rate in the disengagement task (*r* = 0.85) and adequate for mean dwell time and proportion of correct refixations in the disengagement task (*r* = 0.55 for both variables). For latency in the disengagement task and mean dwell time in the face task, the correlations were slightly below the cut-off of 0.50 (i.e., *r* = 0.46 and 0.49, respectively). For transition rate in the face task, the test-retest reliability was low with a correlation of −0.07.

#### Alerting

For the alerting variables, test-retest reliability was good for total dwell time in the alerting task (*r* = 0.79) and total dwell time in the delayed response task (*r* = 0.86). Test-retest reliability was adequate for total dwell time in the face task (*r* = 0.53) and slightly below cut off for total dwell time in the disengagement task (*r* = 0.49). For latency difference in the alerting task, test-retest reliability was low with a correlation of 0.21.

#### Executive Attention

Test-retest reliability was good for number of correct searches in the delayed response task (*r* = 0.71) and adequate for mean delay in the delayed response task (*r* = 0.63).

### Discussion

In this study, test-retest reliability of the UTATE was examined by studying the relationship of the variables from the tasks that underlie the three latent factors, orienting, alerting, and executive attention. Results of the current study showed for the orienting measures adequate to good reliability for 3 out of 6 variables, reliability slightly below cut off for two variables and low reliability for one variable. For the alerting measures, adequate to good reliability was found for 3 out of 5 variables, slightly below cut off for one and low for one variable. Reliability was adequate to good for both variables that measure executive attention.

The goal of this study was to get additional information regarding reliability of the UTATE, next to the previously examined split-half reliability (De Jong et al., 2015, 2016b). This is needed as split-half reliability is not the best method to investigate reliability for every variable, for example because of lack of variation (i.e., proportion of correct refixations) and the inability to make an appropriate split (i.e., mean delay in the delayed response task). This suggestion is supported by the finding that for both of these variables test-retest reliability was adequate to good. When information from split-half reliability and test-retest reliability are combined, reliability was adequate to good for 5 out of 6 orienting measures. For latency in the disengagement task, test-retest reliability was slightly below cut off and split-half reliability was moderate. This indicates that the measure of orienting is a reliable measure, as the reliabilities of the variables from which this measure is constructed were mostly adequate to good. For the alerting measure, the same can be concluded as reliabilities were adequate to good for 4 out of 5 measures.

Reliability was low for one measure underlying the orienting factor, transition rate in the face task. As the means for this variable were almost the same on both measurement occasions and the standard deviation were not large, the variation in scores may have been too small to show a clear relationship over time. Reliability was also low for latency difference in the alerting task and this variable previously was found to show a very small factor loading (De Jong et al., 2016a). The alerting system is thought to reflect the ability to achieve and maintain a state of alertness (Posner and Petersen, 1990). Whereas most variables of the alerting system especially reflect sustained attention, latency difference specifically reflects the ability to achieve a state of alertness. The variation in achieving a state of alertness at two different measurement moments, apparently is large and it seems to differ from the ability to maintain it at both times. As we previously found that an extra analysis with the variables with non-significant factor loadings excluded, also resulted in a model with good fit indices, we have kept these variables in our models for theoretical reasons and it was expected that this had little influence on the measure of alerting (De Jong et al., 2015). For further study of the attention capacities of toddlers the latent factors are considered to be of greater importance than the variables of all tasks separately.

Finally, the executive attention measure can be considered reliable as reliabilities of both variables were adequate to good.

Limitation of the current study is the small sample size (*n* = 12). Although in a previous study we found almost similar results regarding split-half reliability in a pilot sample of 16 and the full sample of 196 children (De Jong et al., 2015, 2016b), further research is needed with larger samples to confirm our findings with respect to test-retest reliability.

In sum, reliability was adequate to good on at least one of the two methods (i.e., split-half reliability and test-retest reliability) for 11 out of 13 variables of the UTATE. This study showed that a combination of different types of reliability assessment provides a more complete picture of the reliability of an instrument, as one type of reliability measure does not suite every variable. For further studies we suggest to use the three latent factors of orienting, alerting and executive attention capacities and not the separate variables of the tasks that constitute those factors. Based on our findings we conclude that, overall, the UTATE is a reliable instrument.

## Study 2 – Convergent and Predictive Validity

### Research Question

To what extent are the results of the UTATE related to other measures of attention at 18 months of age and to attention and cognitive functioning 6 months later at 24 months of age?

### Materials and Methods

#### Participants and Procedure

This study is part of an ongoing longitudinal project on the development of preterm children, the STAP Project (i.e., Study on Attention of Preterm children). The original sample size calculations were based on the possibility of detecting group differences of at least 0.5 standard deviation, while taking into account potential attrition over the years. With a power of 0.80 and an alpha set at 0.05, this analysis showed that at least 64 children should participate in each group.

Here, it concerns the term born children with a gestational age of ≥37 weeks, born in four hospitals in and around Utrecht. Their parents were invited by letter from their midwives when their child was 10 months old, to participate in the study. The participating children were all born between March 2010 and April 2011. Exclusion criteria were dysmaturity [i.e., birth weight below 10th percentile according to Dutch reference curves from Stichting Perinatale Registratie Nederland (now Perined)^1^ ], multiple births, admission to a tertiary Neonatal Intensive Care Unit, severe congenital malformations, antenatal alcohol or drug abuse by the mother, and chronic antenatal use of psychiatric drugs by the mothers.

When the children were 18 and 24 months of age, the mothers were asked to answer questionnaires concerning the development and behavior of their children and their parenting behavior. When the children were 18 months, they visited our lab for an evaluation of attention capacities by means of an eye tracking procedure and an observation of mother-child interaction. At 24 months of age (Wave 2), the children and their mothers visited our lab or the hospitals where they were born for a developmental assessment. The visits were planned in such a way that these would not interfere with the children’s sleeping schedules. The eye tracking procedure is described in detail in De Jong et al. (2016b). After the eye tracking procedure, the mothers were asked to play with their child for 15 min: 5 min of free play and 10 min of structured play (i.e., reading a book and making a puzzle, both for 5 min). The interaction was videotaped and coded afterward.

The research project was approved by the Medical Ethical committee of the University Medical Centre Utrecht. All parents gave informed consent for their child’s participation. At the end of the visits for each wave the child received a present. Parents were reimbursed for their travel expenses.

The sample reported on consist of the 95 term born children who had data available for the UTATE at 18 months of age. They were Dutch children, 44.2% boys, aged around 18 months (*M* = 17.54, *SD* = 0.50) at Wave 1, and 24 months (*M* = 23.85, *SD* = 0.46) at Wave 2. Complete data were available for 89 children. Missing data appeared for the ASQ communication dimension for one girl; one boy and two girls could not be assessed with the Bayley-III-NL and four children (three girls and one boy) did not have ECBQ data available. In total six children (two boys and four girls) had some missing data, and they did not differ from the other 89 children: *F*(2, 92) = 0.855, *p* = 0.429, partial η^2^ = 0.018, in gestation at birth [40.0 (0.60) vs. 39.1 (1.01) weeks], nor in birth weight [3645 (482) vs. 3569 (458) grams]. The parents of the participating children described them as healthy and did not express specific worries about their functioning.

#### Measures

##### Attention Capacities

###### UTATE

The UTATE was administered in a lab setting when the child was 18 months of age, see description above. In this study, the scores on the 13 variables of the UTATE were reduced to scores on three latent constructs (i.e., orienting, alerting, and executive attention). The scores on these latent constructs were computed using the model described in De Jong et al. (2016a). For all constructs, higher scores were considered to be indicative of better attention skills.

###### Mother-child interaction

Mother-child interaction was observed when the child was 18 months old during both a free play (i.e., 5 min) and structured play setting (i.e., 10 min). Regarding the child’s attention behavior during mother-child interaction the on-task persistence subscale of the Coding Interactive Behavior system (CIB; Feldman, 1998), was coded by independent coders who were trained by a certified CIB user (MdJ). On-task persistence is defined as the persistence of a child to continue one task until it is finished and was coded separately during the free play and structured play situation. Scores ranged from 1 to 5, with 1 being a low level and 5 being a high level of on-task persistence. Interrater reliability was good with an intra class correlation of 0.76 based on 21% double coded videos. Validity of the CIB has been confirmed with multiple samples (e.g., Feldman and Klein, 2003).

###### Early Childhood Behavior Questionnaire (ECBQ)

The attention focusing and attention shifting subscales of full version of Early Childhood Behavior Questionnaire (ECBQ; Putnam et al., 2006) and the effortful control scale of the very short version of the ECBQ (ECBQ-VSF) were answered by the mothers when the child was 18 and 24 months of age. The attention focusing (e.g., “When playing alone, how often did your child become easily distracted?”), attention shifting (e.g., “After having been interrupted, how often did your child return to a previous activity?”) and effortful control (e.g., “When told ‘no’, how often did your child stop the forbidden activity?”) subscales consist of 12 questions each with scores ranging from 1 to 7. One indicates that a child “never” exhibited the behavior referred to in the question during the last 2 weeks and seven indicates “always.” A high score on the subscales indicates that a child has better attentional abilities. The ECBQ scales were reliable at both ages (Cronbach’s α = 0.68–0.83).

##### Communication skills and social emotional development

###### Ages and Stages Questionnaires (ASQ)

The communication scale of the Ages and Stages Questionnaire (ASQ; Squires and Bricker, 2009) was used when the toddlers were 18 months of age. The scale consists of six items (e.g., “When your child wants something, does she tell you by pointing to it”), with answering categories “yes,” “sometimes,” and “not yet.” A high sum score on the six items means better skills. The ASQ has been found valid and reliable (Squires and Bricker, 2009; Gollenberg et al., 2010).

###### Ages and Stages Questionnaire – Social-Emotional (ASQ-SE)

The ASQ-SE was filled out by mothers when their child was 18 months of age. The ASQ-SE asks about the social emotional development of the child (e.g., “Does your child like to be around other children”) using 29 items with answering categories “most of the time,” “sometimes,” and “rarely or never.” A sum score on the 29 items is used as measure of social-emotional functioning, with lower scores indicating better functioning. The questionnaire has been found valid (Squires et al., 2002).

#### Developmental Level

##### Bayley-III-NL

At 24 months of age the Dutch version of the Bayley-III, the Bayley-III-NL (Bayley, 2006; Van Baar et al., 2014), was used to assess the developmental level of the children. The Bayley-III –NL consists of five subtests: Cognition, Fine Motor, Gross Motor, Receptive Communication, and Expressive Communication. In the current study, only the score on the Cognition subtest was used. The Cognition subtest is intended to measure sensorimotor development, exploration and manipulation, object relatedness, concept formation and memory. Items include, for example, searching for a hidden object and making puzzles. The index score based on Dutch norms was used, which has a mean of 100 and a SD of 15 (Van Baar et al., 2014). The reliability and validity of the Bayley-III-NL is good with 0.87 for the cognition index and 0.90 for both the language and motor index scores (Van Baar et al., 2014).

##### Statistical analyses

To assess the relationships between the three attention systems as measured by the UTATE and the other measures, Pearson product-moment correlations were computed. In line with Cohen’s (1988) standard, correlations between 0.10 and 0.29 are interpreted as a small association, between 0.30 and 0.49 as a medium association, and above 0.50 as a large association. Predictive validity was also explored in a subgroup of children with low scores on the UTATE, reflected by a score that was more than one standard deviation below the mean, in order to evaluate the potential for identifying children with difficulties in attention. It was studied if this subgroup with low scores differed from the other children on mother-reported attention measures and the cognition scale of the Bayley-III-NL at 24 months of age according to a one-way analysis of variance. Effect sizes are presented as partial η^2^, with values of ≤0.02 seen as small, 0.02–0.13 as medium and ≥0.26 as large (Draper, 2020). SPSS version 25.0 was used for the analyses and alpha was set at 0.05.

### Results

#### Orienting

##### Convergent validity

The orienting system as measured by the UTATE was found to be significantly related to mother-reported attention shifting, *r* = 0.21, *p* = 0.048, with a small effect size.

##### Divergent validity

The orienting system of the UTATE was not related to mother-reported communication skills, *r* = 0.02, *p* = 0.85. A small association, but not significant, was found between the UTATE and social-emotional skills, *r* = −0.18, *p* = 0.09.

##### Predictive validity

No correlation was found between orienting system of the UTATE at 18 months of age and mother-reported attention shifting, *r* = 0.09, *p* = 0.40, and a small, but not significant correlation was found for cognitive functioning, *r* = 0.19, *p* = 0.06 at 24 months of age for the total group. When comparing the subgroup with low scores for orienting on the UTATE (*n* = 10) with the subgroup with normal scores (*n* = 85), a significant difference with a medium effect size was found on cognitive functioning with the subgroup with low scores on the UTATE performing worse than the subgroup with normal scores on the UTATE, *F*(1, 91) = 7.15, *p* = 0.01, partial η^2^ = 0.07 (see Table 3). No differences were found between the subgroups on mother-reported attention shifting at 24 months of age, *F*(1, 89) = 0.21, *p* = 0.65, partial η^2^ = 0.002.

**TABLE 3:** Subgroups of children with low or normal scores on the UTATE attention systems and their mother-reported attention skills and outcome at the Bayley-III-NL Cognition scale.

	Low scores			Normal scores					
	*n*	Mean	*SD*	*n*	Mean	*SD*	*F*	*p*	Partial η^2^
**Mother-reported attention**									
Orienting	8	4.63	0.58	83	4.73	0.56	0.21	0.65	0.002
Alerting	11	4.36	0.80	80	4.50	0.71	0.38	0.54	0.004
Executive attention	12	5.01	0.50	79	4.97	0.64	0.04	0.85	0.000
**Cognitive functioning**									
Orienting	10	95.10	6.10	83	104.67	11.09	7.15	0.01	0.07
Alerting	13	97.77	7.13	80	104.60	11.31	4.43	0.04	0.05
Executive attention	12	101.17	8.84	81	104.01	11.34	0.69	0.41	0.01

*One-way analysis of variance was used; of the 25 children with low scores on at least one of the three attention systems of the UTATE, 16 (i.e., 64%) had a low score on one, 7 (i.e., 28%) on two, and 2 (i.e., 8%) on all three attention systems.*

#### Alerting

##### Convergent validity

The alerting system as measured by the UTATE was significantly related to observed on-task persistence in a structured play setting, *r* = 0.25, *p* = 0.02, with a small effect size. No relations were found between the alerting system of the UTATE and both mother-reported attention focusing and observed on-task persistence in a free play setting.

##### Divergent validity

The alerting system of the UTATE was not related to mother-reported communication skills, *r* = −0.05, *p* = 0.64. A small and not significant correlation was found between the UTATE and social-emotional skills, *r* = −0.18, *p* = 0.08.

##### Predictive validity

Small and not significant correlations were found between the alerting system measured by the UTATE at 18 months of age and both mother-reported attention focusing, *r* = 0.19, *p* = 0.08, and cognitive functioning, *r* = 0.18, *p* = 0.08, at 24 months of age. When comparing the subgroup with low scores for alerting on the UTATE (*n* = 14) with the subgroup with normal scores (*n* = 81), a significant difference with a small to medium effect size was found on cognitive functioning with the subgroup with low scores on the UTATE performing worse than the subgroup with normal scores on the UTATE, *F*(1, 91) = 4.42, *p* = 0.04, partial η^2^ = 0.05 (see Table 3). No differences were found between the subgroups on mother-reported attention shifting at 24 months of age, *F*(1, 83) = 0.38, *p* = 0.54, partial η^2^ = 0.004.

#### Executive Attention

##### Convergent validity

No relation is found between the executive attention system measured with the UTATE and mother-reported effortful control, *r* = 0.03, *p* = 0.78.

##### Divergent validity

The executive attention system of the UTATE was not related to mother-reported communication skills, *r* = −0.13, *p* = 0.22, and not to social-emotional skills, *r* = −0.02, *p* = 0.86.

##### Predictive validity

Executive attention measured with the UTATE at 18 months of age was not related to mother-reported effortful control, *r* = −0.02, *p* = 0.86, and cognitive functioning, *r* = 0.05, *p* = 0.63, at 24 months of age. Comparing the subgroup with low scores for executive attention on the UTATE (*n* = 12) with the subgroup with normal scores (*n* = 83) showed no significant difference on both cognitive functioning, *F*(1, 91) = 0.69, *p* = 0.41, partial η^2^ = 0.01, and mother-reported effortful control, *F*(1, 83) = 0.04, *p* = 0.85, partial η^2^ = 0.000 (see Table 3).

### Discussion

This study concerned the convergent, divergent and predictive validity of the recently developed UTATE, intended to measure the orienting, alerting and executive attention systems of toddlers. For the *orienting* system of the UTATE, convergent validity was confirmed by the significant correlations between the UTATE and mother-reported attention shifting, although this was only a weak relationship. Divergent validity, which was accepted when the correlations were not significant, or showed less strong relationships between instruments supposed to measure different constructs, was partly confirmed as the correlation between orienting and communication and between orienting and socio-emotional skills was not significant. Furthermore, important evidence for predictive validity was found as a low score on the UTATE was predictive of lower scores on cognitive functioning 6 months later. This relationship was not seen in the continuous correlations between the UTATE and the Bayley scores, but it became clear when the results of children with low scores vs. those with better scores on the UTATE, were compared regarding their Bayley outcome results. The subgroup differences appeared to be even 9.5 index points, more than half a standard deviation on the Bayley-III-NL, the golden standard in measuring child development.

The *alerting* system of the UTATE was positively related to observed on-task persistence during a structured play setting. No relation was found with observed on-task persistence during a free play setting and mother-reported attention focusing. Therefore, convergent validity was partly confirmed. Divergent validity for the alerting system was also partly confirmed as the correlation between the UTATE communication was not significant and also the correlation with socioemotional skills was non-significant. As with the *orienting* system, evidence for predictive validity was found as a low score on the *alerting* system of the UTATE was predictive of an almost 7 points lower average in cognitive functioning 6 months later, again showing a relation between the UTATE and the Bayley-III-NL.

Although we did find evidence for convergent validity for the *orienting* and *alerting* system, the effect sizes were relatively small. This might be due to differences in the types of instruments used in the comparison. For example, parental questionnaires are not always reliable in capturing the subtle differences in such domains as visual orienting and this could reduce, in turn, the convergent validity with an eye-tracking measurement of visual orienting, which can be seen as more rigorous. In addition, while the UTATE consists of computerized tasks performed in a lab situation, the questionnaires concerned behavior in a home-setting judged by the mother over a longer period of time. The observations (i.e., alerting system) were done by an independent observer, but measured in a lab setting at one moment. This might result in measuring functioning of different types of attention. Unfortunately, as yet there seems to be no alternative instrument that measures functioning of the orienting and alerting system in this age group in the same way as the UTATE that could be used for validation, which was the main reason for developing the UTATE. For older children, computerized tasks to measure functioning of the three attention systems do exists, such as the child version of the attention network test (child-ANT) for children above 6 years of age (Rueda et al., 2004). To further confirm validity, future research might investigate how performance on the UTATE is related to later performance on computerized tests that measure attention capacities, such as the child version of the Attention Network Test (child-ANT; Rueda et al., 2004), the Early Childhood Attention Battery (ECAB; Breckenridge et al., 2013), or the COTAPP (Rommelse et al., 2016).

For both *orienting* and *alerting*, divergent validity was partly confirmed, as there was no relation with communication skills, but a small relation with social-emotional skills. As already mentioned in the introduction, it is difficult to find developmental constructs that are not related to attention capacities. The small relation between the UTATE and social-emotional skills might have to do with the fact that one of the domains that is measured is self-regulation, of which attention capacities are part of (e.g., Rothbart et al., 2006).

For the *executive attention* system, support of divergent validity was found as the *executive attention* system of the UTATE was not related to both communication and social-emotional skills. No support was found for convergent and predictive validity. To investigate convergent validity, we compared the UTATE to mother-reported effortful control. Although previous research suggested that this temperamental dimension is closely related to executive attention (Rothbart et al., 2007), it is not exactly the same. There are, however, as far as we know, no other measures of executive attention that could be used to confirm convergent validity in this age group. For slightly older children, aged above 2 years, tasks to measure executive functioning do exists and might be used in future research to confirm validity of the executive attention measure of the UTATE.

In sum, in this study different attention capacities as measured with the UTATE that uses eye tracking of looking behavior, were compared to outcomes based on other instruments like mother-reported questionnaires, observations and test results. The findings showed some initial support for convergent, divergent, and predictive validity for the *orienting* and *alerting* system measured with the UTATE, and for divergent validity of the *executive attention* system.

## Study 3 – Attention Measured With the UTATE at Different Ages

### Research Question

Is the UTATE feasible for 12- and 24-month-old children? And are there differences in performance of 12-, 18-, and 24-month-old children? Some first information is presented here on partially small samples, in order to explore these research questions and to provide some first guidance for future studies.

### Materials and Methods

#### Participants and Procedure

The participating children for this study who were aged around 12 months and who were aged around 24 months, were acquired by students in their own network, who asked their parents to collaborate. This study consisted of one assessment with the UTATE and answering a short questionnaire on demographic background characteristics. For the age group at 18 months, data of the UTATE were used from study 2, as described above. Parents and caretakers considered the children to be healthy at the time of assessment. The sample consisted of 14 children aged around 12 months (*M* = 12.07, *SD* = 0.73, 50.0% boys), 95 children aged around 18 months (*M* = 17.54, *SD* = 0.50, 44.2% boys), and 15 children aged around 24 months (*M* = 23.67, *SD* = 0.49, 80% boys).

The research project was approved by the Medical Ethical committee of the University Medical Centre Utrecht. All parents gave informed consent for their child’s participation. At the end of the lab visit the child received a present. Parents were reimbursed for their travel expenses.

#### Measurement Instruments

##### Feasibility

Not only the fact if it was possible to get eye tracking data with the UTATE but also the qualitative circumstances of this measurement for the toddlers themselves were considered important. Feasibility was examined by a qualitative observation of the video tapes of the 12- and 24-month-old children’s faces and behavior during the UTATE procedure. One of us (MdJ) watched all videotapes and gave a description of the children’s behavior. She described whether calibration could be done, to what extent the children stayed seated and moved in their seats, whether they talked or got fuzzy or cried or needed comforting, whether they actually looked at the screen, whether they remained in the car seat or needed to be placed on their caretakers lap. Next to this qualitative impression we checked whether the children could actually provide data for all four tasks of the UTATE.

##### UTATE

The UTATE was administered when the child was 12, 18, or 24 months of age, see description above (Study 1).

#### Data Analysis

To examine differences in performance of the three age groups, first the graphical display of the mean scores per age group will be inspected to investigate whether the expected improvement in scores is shown, because of the relatively small sample sizes in the 12- and 24-month-old groups. Additionally, the performances of the three groups of children are compared using three univariate analyses of variances (ANOVAs) for the three latent constructs. Effect sizes are presented as partial η^2^, with values of ≤0.02 seen as small, 0.02–0.13 as medium and ≥0.26 as large (Draper, 2020). LSD *post-hoc* analyses are used to investigate which of the groups statistically differs from each other.

### Results

#### Feasibility: Cooperation of the Children and Circumstances During the UTATE

Of the 12-month-old children, all 14 provided data on all four tasks. In the group of 24-month-olds, data on all four tasks was available for 13 of the 15 children. The two children with incomplete data both missed data on two tasks due to refusal to participate.

There were no cases of calibration failure.

During the UTATE procedure, children were preferably placed in a car seat to constrain them somewhat in their movements. Three 24-month-old children refused to sit in the car seat beforehand and were placed on their parents’ lap. Two children, one 12-month-old and one 24-month-old, changed position (i.e., from car seat to parents’ lap) during the procedure because of refusal to sit in the car seat: one child between the disengagement and face task, and one child between the face and alerting tasks.

Viewing the video recordings showed that both 12- and 24-month-old children generally sat at ease, looked at the screen with interest most of the time, moved a bit with the sounds and sometimes looked at their parents. All in all, most children showed to be able to cooperate and they looked at the tasks frequently enough to provide data for calibration and task performance.

#### Performance on the Tasks

**FIGURE 1:**
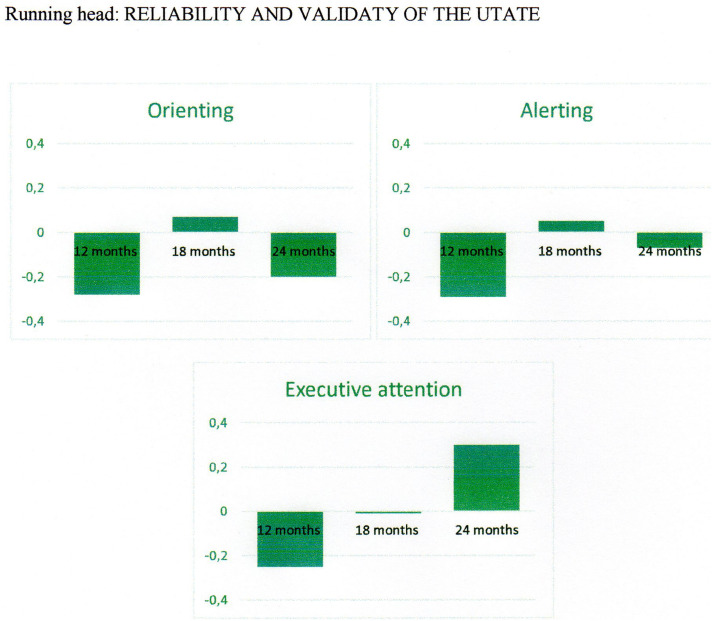
Scores on the latent constructs per age group.

The means and standard deviations on the variables are presented per age group in Table 4 and graphically in Figure 1.

**TABLE 4:** Means and standard deviations on the UTATE variables for the separate age groups.

	12 months (*n* = 14)	18 months (*n* = 95)	24 months (*n* = 15)
	Mean (*SD*)	Mean (*SD*)	Mean (*SD*)
Orienting	−0.28(0.87)	0.07(0.85)	−0.20(0.72)
Alerting	−0.28(0.87)	0.05(0.87)	−0.07(0.80)
Executive attention	−0.25(1.00)	−0.01(1.10)	0.30(0.76)

##### Orienting

Figure 1 shows that 18-month-olds performed (slightly) better on orienting than both 12- and 24-month-olds. The latter two groups showed a similar performance. These differences were not statistically significant, *F*(2, 121) = 1.53, *p* = 0.22, partial η^2^ = 0.03.

##### Alerting

For alerting, Figure 1 shows less alerting for children of 12 months than for children of 18 months of age, but no difference between children of 18 and 24 months of age. As with orienting, the differences between the groups were not statistically significant, *F*(2, 121) = 0.96, *p* = 0.39, partial η^2^ = 0.02.

##### Executive attention

Children of 12 months showed less executive attention than children at 18 months and they showed less executive attention than children of 24 months of age, see Figure 1. These differences were, however, not statistically significant, *F*(2, 121) = 1.01, *p* = 0.37, partial η^2^ = 0.02.

### Discussion

In this study, a first impression was given of the feasibility of the UTATE in 12- and 24-month-old children and the potential to detect age differences in orienting, alerting and executive attention capacities between 12, 18, and 24 months of age. Together with our previous study on feasibility in 18-month-old children (De Jong et al., 2016b), results showed that the UTATE is feasible for 12-, 18-, and 24-month-old children, as most of the children cooperated quite well. Regarding age differences, for alerting attention, lower scores were seen at 12 months, compared to 18 months of age, and stable scores between 18 and 24 months of age. For orienting attention lower scores were seen for children of 12 months compared to children of 18 months, but scores of children at 24 months of age showed around the same level of performance as 12-month-olds. The differences between the age groups were not significant, which may be explained by the small number of children studied at 12 and 24 months.

The results of this study seem to indicate different levels of functioning at different ages for the different attention systems. Although we hypothesized that better performance would be expected for all three attention systems for children of older ages (Ruff and Rothbart, 1996), the age differences in our study showed a distinct pattern. Despite previous findings that the orienting and alerting system continue to develop at least until childhood (e.g., Rueda and Posner, 2013), functioning also has been found to improve already largely during the first year of live (Ruff and Rothbart, 1996). Executive attention, on the other hand, starts to develop at the end of the first year of life (Ruff and Rothbart, 1996). It might therefore be that functioning of the orienting and alerting system is fairly stable for children at different ages during the second year of life, while executive attention shows better scores for older children. It is possible that the development of executive attention interferes with orienting performance. Orienting is the ability to activate attention and shift between targets (Posner and Petersen, 1990; Atkinson and Braddick, 2012). This is mainly driven by external cues (Ruff and Rothbart, 1996). Executive attention, on the other hand, is more internally driven and includes control of attention and inhibition of behavior (Colombo, 2001; Atkinson and Braddick, 2012). When a child is starting to use more attentional control and behavioral inhibition, this might result in less optimal orienting strategies, as those are more spontaneous or reflexive. Of course, it is too early to draw conclusions about our results for children in different ages groups based on the small samples and the cross-sectional design of this study, but the findings do point to the importance of further studying the development of orienting, alerting, and executive attention during the first years of life.

All children in the three samples at 12, 18, and 24 months were considered by their parents to be healthy and their parents expressed no specific concerns. However, we did not evaluate the children’s cognitive abilities in these subgroups in detail, except for using the UTATE, which can be seen as a limitation of this study. In addition, due to the small sample size, there was a lack of statistical power to find statistically significant differences. However, the goal of this study was to get a first impression of the feasibility of the UTATE at 12, 18, and 24 months of age and the ability of the UTATE to detect differences between age groups. Based on the findings of this study, further studies with the UTATE could be useful for acquiring information on early attention development.

## Conclusion

The studies on the UTATE, which was intended to measure functioning of the orienting, alerting, and executive attention systems of toddlers, showed some first promising evidence for test-retest reliability, extending the previously reported split half reliability (De Jong et al., 2015, 2016b). In addition to previous evidence for factorial and clinical validity of the UTATE (De Jong et al., 2015, 2016a) some first preliminary indications of convergent, divergent, and predictive validity of the UTATE were found. However further studies are needed in view of some small sample sizes used, specifically for evaluation of the results and use of the UTATE at younger and older ages than 18 months.

## Data Availability Statement

The datasets generated for this study are available on request to the corresponding author.

## Ethics Statement

The Medical Ethical Committee of the Utrecht Medical Center, Utrecht, Netherlands approved this study. Written informed consent to participate in this study was provided by the participants’ parents or legal guardian.

## Author Contributions

AB conceptualized and designed the study, reviewed and revised the manuscript, and approved the final manuscript as submitted. MV designed the study, wrote the first version of the manual, reviewed and revised the manuscript, and approved the final manuscript as submitted. MJ designed the study, performed the data collection, carried out the analyses, revised the manuscript, and approved the final manuscript as submitted. MM carried out the analyses, drafted the initial manuscript, revised the manuscript, and approved the final manuscript as submitted. IH prepared the eye-tracking data for analyses, assisted in data analyses, reviewed and revised the manuscript, and approved the final manuscript as submitted. LB reviewed and translated and wrote the final version of the manual, revised the manuscript, and approved the final manuscript as submitted.

## Conflict of Interest

The authors declare that the research was conducted in the absence of any commercial or financial relationships that could be construed as a potential conflict of interest.

## Footnotes

1 https://www.perined.nl/gegevensgebruik/publicaties/geboortegewichtcurven (accessed May 23, 2020).
