# Supplementary material for: Reliability and Validity of the Utrecht Tasks for Attention in Toddlers Using Eye Tracking (UTATE)
Source: Front Psychol. 2020 Jun 8;11:1179. doi: 10.3389/fpsyg.2020.01179 (PMC7325908; doi:10.3389/fpsyg.2020.01179)
Supplement: Supplementary file 1 [file Data_Sheet_1.pdf]

Supplement

UTATE

UTRECHT TASK OF ATTENTION IN TODDLERS USING EYE TRACKING

Manual

Marjanneke de Jong
Lilly Bogičević
Marjolein Verhoeven
Anneloes van Baar

Utrecht University
2011-2020

Utrecht University

1 – APPARATUS

Eye tracker: Tobii T60 Eye Tracker with integrated 17-inch TFT screen with a resolution of 1280 x 1024 pixels.
Software for presenting stimuli on the screen: E-prime 2.0.

2 – LAB SITUATION

An almost dark and soundproofed room. A lightbulb oriented towards the ceiling is used to make the room less dark, and less frightening for children, without distorting the eye tracking measures.

The child is placed into a car seat to keep them in a sitting position and somewhat constrain their movements, so that the eye tracker can follow their eye movements properly. The car seat should be positioned at ±65 cm from the eye tracker.

The examiner is seated at a desk with the test computer, behind a curtain preventing the child from seeing the examiner.

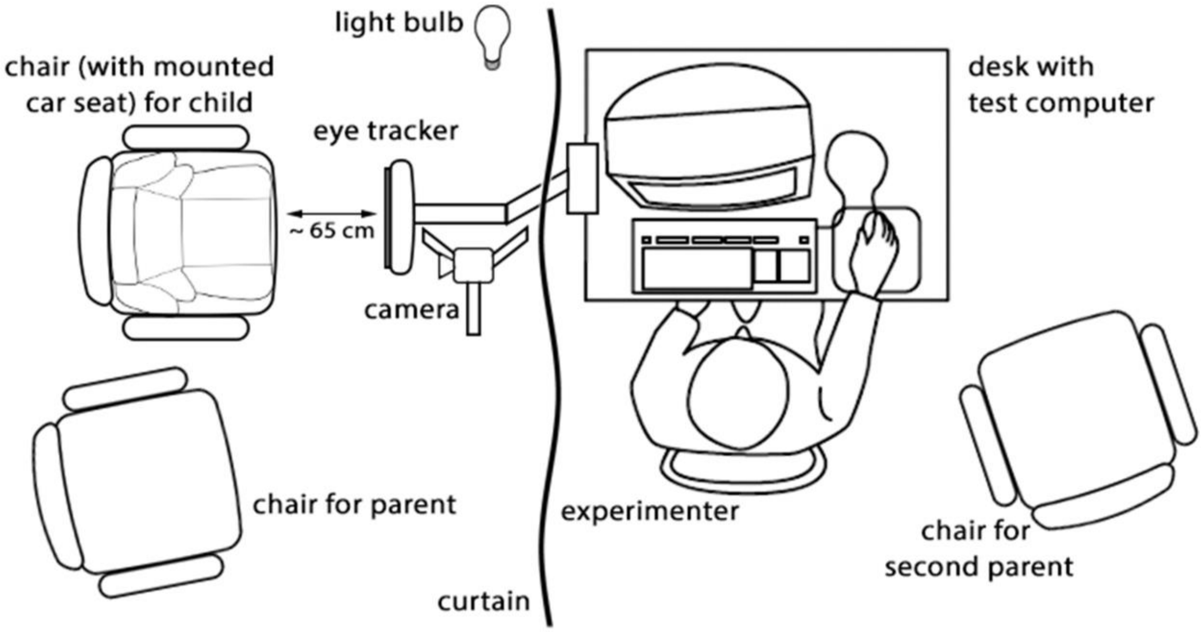

3 – EYE TRACKER TASKS

The eye tracker task consists of four different tasks: the Disengagement Task, the Face Task, the Alerting Task, and the Delayed Response (DR) Task. The experiments for these tasks can be provided for the software E-prime 2.0 and may be used on the same apparatus. Because properties may vary per apparatus, the experiments may not work properly on other eye trackers and different screen sizes. In this case, the stimuli used in the tasks can be provided in order to remake the experiments in either E-prime or other software and suited for the available apparatus.

The tasks are described in detail by De Jong, Verhoeven, Hooge & Van Baar (2016).[1]

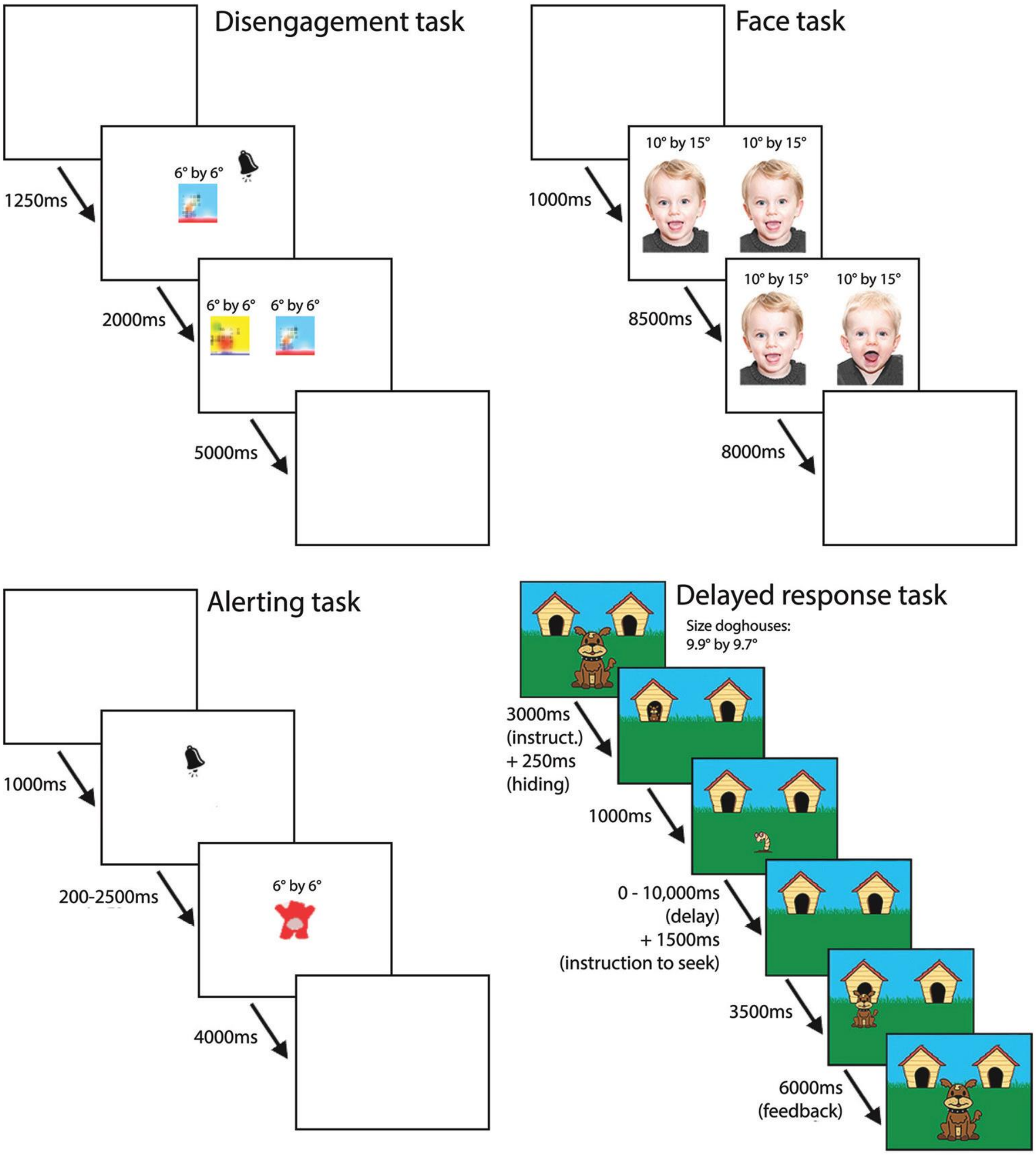

Visualization of timing and size of the stimuli in the different tasks

4 – ASSESSMENT PREPARATION

<u>4.1 Preparing eye-tracker room</u>

- Turn on computer + Tobii (eye-tracker).
- Take lab form and fill in ID number of participant.
- Place camera on tripod.
    - Position camera on the tripod to the right behind the eye-tracker and make sure that the camera is positioned over the eye-tracker. Focus camera on the child's face (flower on the car seat serves as indication).

- Position the car seat at op ±65 cm from the eye tracker and make sure the straps are folded outward so the child can sit in the car seat.

- Turn on the light next to the curtain and turn off all other lights. Check whether the curtain is closed properly.

4.2 Preparing the eye-tracker

- Start Tobii Studio on the computer for calibrating.
    - Check calibration settings
        - Calibration_UTATE[1].
        - Setup → settings → tab - calibration.
            - Infant
            - Animation = bouncing ball
            - Animation 2 = 5.avi
            - 9 dots
            - Background color = white
            - OK.
    - Add new participant:
        - Setup → manage participants → new → enter ID code participant → OK → OK.

- Prepare 4 experiments:
    - UTATE folder[2] → open from the folders 1 t/m 4. The experiments are 'Disengagement', 'Face Task', 'Alerting', and 'DR Task'. Open the files with the extension *.es2*. Wait until each experiment is fully loaded before opening the next experiment to ensure the experiments will run in the right order. Open the experiments in full screen.

- Start Gimp 2 [3]for saving a screenshot of the calibration:
    - File → new → width: 1280, height: 1024 → OK.

- Make sure Tobii Studio is open on the screen before picking up the participant:
    - Setup → calibrate → tab: use existing participant → choose ID code of participant → continue.
    -

5 – ASSESSMENT

---

[1] Tobii Studio allows you to make a standard setting and name it, we named it Calibration_UTATE. Alternatively, it's possible to just click the settings mentioned below each time at set-up.

[2] Add path to the folder you will use.

[3] We used the image software program Gimp 2 to save screenshots of each participants calibration, so we could later check the calibrations. Tobii Studio does not save the calibration. Other image software programs can also be used.

5.1 Welcome mother/parents and child[4]:

5.1.1 Instructions

- Ask mother and child to take a seat at the place where you will welcome them[5].

- Explain what the assessment will entail, e.g.: *"We will go to another room where <name child> will be looking at pictures and videos on a screen. This will take about 15 minutes."*

- Disclaim to mother/parents that we will be making a video, so we can later see whether the child was engaged with the assessment / looking at the screen / etc.

- Ask whether there are further questions and ask mother and child to follow to the eye tracker room.

- If both parents are present, ask them before entering the eye-tracker room which parent will sit next to the child and which parent will take a seat next to you behind the experimenter.

- Ask mother and child to take place in front of the eye-tracker.
    - The child should be seated in the car seat with the straps closed. The mother can sit in the chair next to the car seat. In the case that the child protests or cries and will not sit in the car seat, the child can sit in the mother's lap in the regular chair. Make sure that the chair is positioned in front of the eye-tracker.
    - Position the child at a distance of ± 65 cm of the screen and position the height of the eye-tracker so the child's eyes will be on screen.
    - Turn on the camera and ensure the child's face is visible on camera.

- Give the following instructions to the mother:

"For this task <name child> should sit as calmly as possible. We ask you to talk to <name child> as little as possible. In the case that <name child> asks for a reaction, it is okay if you respond, but you do not need to tell him/her to watch the screen. Do you have any questions? Shall we begin?

- Close the door to the eye tracker room.

5.2 Eye tracker procedure

5.2.1 Eye tracker calibration

- Start calibration in Tobii-studio.
- First attract the child's attention to the screen using the duck animation. Press the upward arrow key for the duck animation.
- Calibrate the 9 dots. Press the space key to move to the next dot. Move to each next dot in 1-2 seconds. If possible, observe through the curtain whether the child is looking at the

[4] Specify your own protocol.

[5] We welcomed the families in another room first instead of going to the dark eye-tracker room directly.

screen. If the child is not looking, press the upward arrow key for the duck animation again.

- After calibrating, check whether the child has looked at ≥ 7 dots.
- Take a screenshot (shift + print screen) of the calibration and paste this in Gimp 2 (Ctrl + V).
- Explain to the mother what the screen is showing:

“These 9 grey dots on the screen show where on the screen the dots were previously shown. The green lines are where your child was looking at. As you can see <name child> did well, so we will continue with the next task.”

- Accept calibration.
- If the child looked at < 7 dots, you need to recalibrate the missing dots. Do this by clicking the missed dots and clicking recalibrate. Start at the beginning of the eye tracker procedure (3.2).

Explain the following to the mother: *“These 9 grey dots on the screen show where on the screen the dots were previously shown. The green lines are where your child was looking at. As you can see <name child> did not yet look at all the dots, so we will do this task again.”*

- Recalibrating may be done with a maximum of 2 times, otherwise the child is likely to lose interest.

5.2.2 Eye tracker experiments

- Start the first experiment ‘Disengagement’.
    - Desktop → open tab Disengagement in E-studio → run experiment→ enter participant ID number → enter → press space key to start.

- Wait until the first experiment is completed and start the next experiment ‘Face Task’.
    - open tab Face Task in E-studio → run experiment→ enter participant ID number → enter → press space key to start.

- Wait until the second experiment is completed and start the next experiment ‘Alerting’.
    - open tab Alerting in E-studio → run experiment→ enter participant ID number → enter → press space key to start.

- Wait until the third experiment is completed and start the last experiment ‘DR Task.
    - open tab DR Task in E-studio → run experiment→ enter participant ID number → enter → press space key to start.

- After the fourth experiment is completed, tell the mother the task is finished and she may take her child out of the car seat.

6 – WRAP UP

6.1 Save assessment:

- Save experiments:
    - <insert file path where experiments will be saved> → In all four experiment folders (Disengagement, Face Task, Alerting and DR task) you will find three files per participant (ID number of the participant).

These files can be recognized by the following extensions: *.txt*, *.gazedata* and *.edat2* → Copy paste these files on a USB stick[6] and/or move them to folder <insert file path of folder used to save eye tracker data>.

6.2 Turn off eye tracker and computer

- Turn off the four experiments in E-studio (do not save the experiments in E-studio).

- Calibration:
  - Save the calibration:
    - File → save as → select USB (left side of the screen) USB → select file type → JPEG image → name → IDnumber-calibration (e.g. 101-calibration) → save → export → save.
  - Turn off calibration:
    - Press the close button twice.

- Eject USB stick safely and take it with you.

- Turn off computer and Tobii (eye tracker).

- Make sure the eye tracker room is left neatly.

7 – DATA EXTRACTION AND PREPARATION

De Jong, et al. (2016)[1] describe the data extraction and preparation in detail. “Matlab 7.11 (The MathWorks, Inc.) was used to analyze gaze data. Fixation detection was done by a self-written Matlab program (I.H.) that marked fixations by an adaptive velocity threshold method. We used an adaptive velocity threshold method to detect fixations because the amount of noise may vary a lot in eye tracking data (especially with low frequency trackers such as the Tobii T60 and with non-grown-up participants). Many modern saccade and fixation detection methods are partly or fully adaptive (Nystrom & Holmqvist, 2010[3]; Smeets & Hooge, 2003[4]). Velocities were obtained by fitting a parabola through three subsequent data points. We used the derivative of this fitted parabola to estimate the value of the velocity of the second (centre) data point. This procedure was repeated for all data points (except the first and the last). In the present analyses, everything that is not a saccade is called a fixation. To remove the saccades from the signal we calculated average and standard deviation from the absolute velocity signal. All data points having absolute velocities higher than the average velocity plus 3 times the standard deviation were removed. This procedure was repeated until the velocity threshold converged to a constant value or the number of repetitions reached 50. Then we removed fixations having durations shorter than 60 ms from the analysis. The value of 60ms was chosen because it is equal to three data samples. When a saccade was removed, the preceding and succeeding fixations were added together. Data of the children were included when they looked at the stimuli at least once during a task, and thereby providing data on the variables of this task.” Thirteen variables were derived from these tasks (De Jong et al., 2016). The whole procedure to do the UTATE takes about 18 minutes.

7.1 File preparation

[6] We saved the experiments on a USB and on the computer to ensure a back up.

- Change file names on the USB stick[7]. ID number should be stated before the task name:
    - E.g. Alerting-001 → 001-Alerting.

- Back-up files[8] <specify where, including folders>.
    - Folders for each task and within, folders for each extension:
        - .edat
        - .txt
        - .gazedata
        - .xml
    - Folder for calibrations

7.2 Fixation detection

Fixation detection was done by a self-written Matlab program, marking fixations by an adaptive velocity threshold method described in detail by Hooge[9] & Camps (2013).[2]

7.2.1 Preparing files for fixation detection by eye tracker expert

- Create a folder with "fixation detection to be processed".
- Add all new files with the extension .gazedata.
- These files should be sent to an eye tracker expert who can process these data using fixation detection.
- The eye tracker expert should return the data after fixation detection in 4 folders (Alerting, Disengagement, DR Task, Face Task).

7.2.2 After fixation detection by eye tracker expert[10]

- Open the folder Alerting → textfiles → alldata.txt.
- Rename alldata.txt file to contain name or abbreviation of task and project, e.g. AL_project.
- Copy this file to location where further data preparation will take place (e.g. folder named 'Variable computation from fixations').
- Repeat the steps above for the other three tasks. Abbreviations often used are AL for Alerting, DIS for Disengagement, DR for Delayed Response Task, FAC for Face Task.

7.3 Variable computation from fixations

After fixation detection, the data represents fixations. These fixations need to be computed into variables however. Fixations can be analyzed using Matlab 7.11.

7.3.1 Preparation for computing variables

- Open folder 'Fixation processor'.
- Move the 4 .txt files to Fixation processor → data.

7.3.2 Fixation processor procedure

[7] We used the files stored on the USB stick for further data extraction and preparation. This may be changed according to your own protocol.

[8] Create your own folders.

[9] We send our gazedata files to our eye tracker expert, who wrote this Matlab program for fixation detection.

[10] This is dependent on the procedure your eye tracker expert will use and should be adjusted accordingly.

Fixations are processed per task to compute the following variables.

- Disengagement Task:
    - mean dwell time
    - transition rate
    - proportion of correct refixations
    - latency
    - total dwell time
- Face Task:
    - mean dwell time
    - transition rate
    - total dwell time
- Alerting Task:
    - latency difference
    - total dwell time
- DR Task:
    - correct searches
    - mean delay
    - total dwell time

De Jong et al. (2016)[1] describe in detail what these variables intend to measure.

Disengagement Task

1. Move event file DIS_project.txt to folder ‘data’ – ‘disengagement’.
2. Run Matlab file ‘DIS_dwelltime.m’. *These are the dwell times per trial per participant.*
3. Run Matlab file ‘DIS_mean_dwell.m’. *These are the dwell times per participant.*
4. Run Matlab file ‘DIS_mean_dwell_split1.m’. *These are the dwell times in trials with even numbers.*
5. Run Matlab file ‘DIS_mean_dwell_split2.m’. *These are the dwell times in trials with uneven numbers.*
6. Run Matlab file ‘DIS_dwelltime_tweedefase.m’. *These are needed for the transition variables.*
7. Run Matlab file ‘DIS_mean_dwell_tweedefase.m’. *These are the transitions per participant.*
8. Run Matlab file ‘DIS_mean_dwell_tweedefase_split1.m’. *These are the transitions in trials with even numbers.*
9. Run Matlab file ‘DIS_mean_dwell_tweedefase_split2.m’. *These are the transitions in trials with uneven numbers.*
10. Run Matlab file ‘DIS_latency_17_10.m’. *These are latencies per trail per participant.*
11. Run Matlabfile ‘DIS_mean_latency17_10.m’. *These are latencies per participant.*
12. Run Matlabfile ‘DIS_mean_latency17_10_split1.m’. *These are latencies in trials with uneven numbers.*
13. Run Matlabfile ‘DIS_mean_latency17_10_split2.m’. *These are latencies in trials with even numbers.*
14. Move event file from folder ‘data’ – ‘disengagement’.

Alerting Task

1. Move event file AL_project.txt to folder ‘data’ – ‘alerting’.
2. Run Matlab file ‘AL_Latency17_10.m’. *These are latencies per trail per participant.*
3. Run Matlab file ‘AL_Mean_Latency.m’. *These are latencies per participant.*

4. Run Matlab file 'AL_Mean_Latency_split1.m'. *These are latencies in the first half of the trials.*
5. Run Matlab file 'AL_Mean_Latency_split2.m'. *These are latencies in the second half of the trials.*
6. Run Matlab file 'AL_dwelltime.m'. *These are the dwell times per trial per participant.*
7. Run Matlab file 'AL_mean_dwell.m'. *These are the dwell times per participant.*
8. Run Matlab file 'AL_mean_dwell_split1.m'. *These are the dwell times in the first half of the trials.*
9. Run Matlab file 'AL_mean_dwell_split2.m'. *These are the dwell times per trial per participant.*
10. Move event file from folder 'data' – 'alerting'.

<u>Face Task</u>

1. Move event file GEZ[11]_project.txt to folder 'data' – 'Faces[12]'
2. Run Matlab file 'GEZ_dwelltime.m'. *These are the dwell times per trial per participant.*
3. Run Matlab file 'GEZ_mean_dwell.m'. *These are the dwell times per participant.*
4. Run Matlab file 'GEZ_mean_dwell_split1.m'. *These are the dwell times in trials with uneven numbers*
5. Run Matlab file 'GEZ_mean_dwell_split2.m'. *These are the dwell times in trials with even numbers.*
6. Run Matlab file 'GEZ_pertrial.m'. *These are the transitions per trial per participant.*
7. Run Matlab file 'GEZ_ntrials.m'. *These are the transitions per participant.*
8. Move event file from folder 'data' – 'Faces'.

<u>DR Task</u>

1. Move event file DR_project.txt to folder 'data' – 'DR'
2. Run Matlab file 'DR_dwelltime.m'. *These are the dwell times per trial per participant.*
3. Run Matlab file 'DR_mean_dwell.m'. *These are the dwell times per participant.*
4. Run Matlab file 'DR_mean_dwell_split1.m'. *These are the dwell times in trials with even numbers.*
5. Run Matlab file 'DR_mean_dwell_split2.m'. *These are the dwell times in trials with uneven numbers.*
6. Run Matlab file 'DR_pertrial17_10.m'. *These are correct searches per trial per participant.*
7. Run Matlab file 'DR_mean17_10.m'. *These are the correct searches per participant.*.
8. Run Matlab file 'DR_mean17_10_split1.m'. *These are correct searches in trials with even numbers.*
9. Run Matlab file 'DR_mean17_10_split2.m'. *These are correct searches in trials with uneven numbers.*
10. Move event file from folder 'data' – 'DR'.

<u>Noise</u>

1. Move the event files AL_noise.xls, DIS_noise.xls, GEZ_noise.xls en DR_noise.xls to the folders 'data' – (name task). Move all event files to the according folders before proceeding.
2. Run Matlab file 'noise17-10.m'.

---

[11] This is the Dutch abbreviation we used for the Face Task (Faces taak in Dutch).

[12] Faces in Dutch. May be adjusted in own files and Matlab code.

3. Move event files from the folders.

<u>Export results to SPSS</u>

1. The folder 'results' should contain output files for each task. These files are tab separated files, which need to be formatted as excel work folders before exporting them to SPSS.

The following files should contain the variables:

a. 'Alerting' – 'AL_mean_dwell'
b. 'Alerting' – 'AL_mean_dwell_split1'
c. 'Alerting' – 'AL_mean_dwell_split2'
d. 'Alerting' – 'AL_meanlatency'
e. 'Alerting' – 'AL_meanlatency_split1'
f. 'Alerting' – 'AL_meanlatency_split2'
g. 'DR' – 'DR_mean_dwell'
h. 'DR' – 'DR_mean_dwell_split1'
i. 'DR' – 'DR_mean_dwell_split2'
j. 'DR' – 'DR_mean'
k. 'DR' – 'DR_mean_split1'
l. 'DR' – 'DR_mean_split2'
m. 'Disengagement' – 'DIS_mean_dwell'
n. 'Disengagement' – 'DIS_mean_dwell_split1'
o. 'Disengagement' – 'DIS_mean_dwell_split2'
p. 'Disengagement' – 'DIS_mean_latency'
q. 'Disengagement' – 'DIS_mean_latency_split1'
r. 'Disengagement' – 'DIS_mean_latency_split2'
s. 'Disengagement' – 'DIS_mean_dwell_transition'
t. 'Disengagement' – 'DIS_mean_dwell_transition_split1'
u. 'Disengagement' – 'DIS_mean_dwell_transition_split2'
v. 'Faces' – 'GEZ_mean_dwell'
w. 'Faces' – 'GEZ_mean_dwell_split1'
x. 'Faces' – 'GEZ_mean_dwell_split2'
y. 'Faces' – 'GEZ_ntrials'
z. 'Alerting_Noise17_10'
aa. 'DR_Noise17_10'
bb. 'Disengagement_Noise17_10'
cc. 'Faces_Noise17_10'

2. Open files in Excel. Sometimes this generates error messages, but these can be ignored. When clicking 'yes', the files will be opened in Excel. The different variable files are not yet merged, because the number of participants may vary across variables. Save each file as 'excel 16-2019 work folder'. Because the noise files have variable names that are the same for each task, the variable names should be changed by adding task abbreviations (e.g. AL, DIS, GEZ, DR). Move all these files to the folder 'excelfiles'.

3. Open SPSS and click 'file' – 'open database' – 'new query'. Select Excel files and import all 29 results files listed above.

4. These 29 files should be merged into 2 files. Because the number of participants may vary across variables, it should be ensured that all the files are merged correctly, e.g. by using an existing file that already contains all participants and their according ID numbers.

a. File 1 contains all eye tracker variables needed for the CFA model and measurement invariance tests. Variables are:

i. 'Alerting' – 'AL_mean_dwell'

ii. ‘Alerting’ – ‘AL_meanlatency’
iii. ‘DR’ – ‘DR_mean_dwell’
iv. ‘DR’ – ‘DR_mean’
v. ‘Disengagement’ – ‘DIS_mean_dwell’
vi. ‘Disengagement’ – ‘DIS_mean_latency’
vii. ‘Disengagement’ – ‘DIS_mean_dwell_transition’
viii. ‘Faces’ – ‘GEZ_mean_dwell’
ix. ‘Faces’ – ‘GEZ_ntrials’

b. File 2 contains all variables needed to assess split half reliability and amount of noise:
   i. ‘Alerting_Noise17_10’
   ii. ‘DR_Noise17_10’
   iii. ‘Disengagement_Noise17_10’
   iv. ‘Faces_Noise17_10’
   v. ‘Alerting’ – ‘AL_mean_dwell_split1’
   vi. ‘Alerting’ – ‘AL_mean_dwell_split2’
   vii. ‘Alerting’ – ‘AL_meanlatency_split1’
   viii. ‘Alerting’ – ‘AL_meanlatency_split2’
   ix. ‘DR’ – ‘DR_mean_dwell_split1’
   x. ‘DR’ – ‘DR_mean_dwell_split2’
   xi. ‘DR’ – ‘DR_mean_split1’
   xii. ‘DR’ – ‘DR_mean_split2’
   xiii. ‘Disengagement’ – ‘DIS_mean_dwell_split1’
   xiv. ‘Disengagement’ – ‘DIS_mean_dwell_split2’
   xv. ‘Disengagement’ – ‘DIS_mean_latency_split1’
   xvi. ‘Disengagement’ – ‘DIS_mean_latency_split2’
   xvii. ‘Disengagement’ – ‘DIS_mean_dwell_transition_split1’
   xviii. ‘Disengagement’ – ‘DIS_mean_dwell_transition_split2’
   xix. ‘Faces’ – ‘GEZ_mean_dwell_split1’
   xx. ‘Faces’ – ‘GEZ_mean_dwell_split2’

5. Sort all SPSS files on ID number in same order. Merge in SPSS by clicking ‘Data’ – ‘Merge files’ – ‘Add variables’ and choose ‘Match cases on key variables in sorted files’ – ‘non-active dataset is keyed table’. Move ID number[13] to ‘key_variables’.

6. Save files as:
   a. File 1 as ‘Eyetrackervars.sav’
   b. File 2 as ‘Eyetracker_noise_split.sav’

<u>7.4 Confirmatory factor analysis to form latent constructs</u>

The variables listed above were used to measure factor scores on three latent constructs: orienting attention, alerting attention, and executive attention. De Jong, Verhoeven, Hooge, & Van Baar (2016b)[3] also describe the confirmatory factor analysis used to confirm these three constructs.

Factor scores can be derived in two ways:

1. In case of a large sample size, measurement invariance may be tested.
2. For smaller sample sizes it is possible to apply the CFA model to new data as used by De Jong et al., (2016)[3], without making changes to the model.

---

[13] Variable containing ID number of each participant

The CFA model is tested in R using Lavaan, which can be download on the internet.
R: https://www.r-project.org/
Lavaan: http://lavaan.ugent.be/

7.4.1 Measurement invariance

1. Measurement invariance can be tested using the SPSS file 'eyetrackervars.sav', to assess whether the CFA model described by De Jong et al (2016)[5] can also be applied to another sample. We tested measurement invariance using the method described by Van De Schoot, Lugtig & Hox (2012) [6].
    a. First the eye tracker variables need to be transformed into the variables needed for the CFA. Open Eerste the SPSS file 'eyetrackervars.sav' and run syntax 'eyetrackervariables.sps'. Save the file with transformed variables.
    b. Ensure the file contains only the variables that are needed for measurement invariance, otherwise participants may be deleted from analysis due to listwise deletion. Variables needed are:
        i. ID number
        ii. Group membership (if applicable)
        iii. AL_total_dwellnosac
        iv. AL_lat_diff
        v. DIS_mean_dwellnosac
        vi. DIS_total_dwellnosac
        vii. DIS_latency
        viii. DIS_transrate
        ix. DIS_prop_correct
        x. DR_ngoed
        xi. DR_meandelay
        xii. DR_total_dwellnosac
        xiii. GEZ_mean_dwellnosac
        xiv. GEZ_total_dwellnosac
        xv. GEZ_transrate

       Save this file as 'eyetrackervars_needed.sav'
    c. Open the measurementinvariance.R file, last used in R version R3.0.2 with the Lavaan package version 05.14. Run this file.
    d. This will create two files:
        i. Modellen_variance.txt → Contains results of the tested models.
        ii. STAP_latent.txt → Contains scores on latent constructs for each participant.
2. The file 'STAP_latent.txt' should be saved as excel file before exporting in SPSS. moet Open this file in excel and save it as excel work folder. Column names may have moved incorrectly, and should all be moved one column to the right. The first colum (without name) can be deleted. Save this file as 'STAP_latent.xls'
3. Import 'STAP_latent.xls' in SPSS.
4. The file 'eyetracker_noise_split.sav' contains split-half variables and noise variables on the eye tracker data. This file can be merged with the SPSS file made from 'STAP_latent.xls', and saved as 'eyetracker_totaal.sav'.

7.4.2 Applying the original CFA model to new data without testing measurement invariance

1. To apply the CFA model described by De Jong et al (2016)[3], first the SPSS file 'eyetrackervars.sav' is needed.
    a. First the eye tracker variables need to be transformed into the variables needed for the CFA. Open the SPSS file 'eyetrackervars.sav' and run syntax 'eyetrackervariables.sps'. Save the file with transformed variables.
    b. Ensure the file contains only the variables that are needed for measurement invariance, otherwise participants may be deleted from analysis due to listwise deletion. Variables needed are:
        i. ID number
        ii. Group membership (if applicable)
        iii. AL_total_dwellnosac
        iv. AL_lat_diff
        v. DIS_mean_dwellnosac
        vi. DIS_total_dwellnosac
        vii. DIS_latency
        viii. DIS_transrate
        ix. DIS_prop_correct
        x. DR_ngoed
        xi. DR_meandelay
        xii. DR_total_dwellnosac
        xiii. GEZ_mean_dwellnosac
        xiv. GEZ_total_dwellnosac
        xv. GEZ_transrate

        Save this file as 'eyetrackervars_needed.sav'
    c. To determine the factor scores based on the original CFA model of De Jong et al (2016)[3], the scores of the term born group of 18 month old infants are needed. The file 'STAP original eyetrackervars_needed.sav' contains these scores.
    d. A separate file with the new participants' data is needed. This file should be named 'eyetrackervars_needed.sav'.
    e. Open file 'Determination_factors_R' last used in R version R3.0.2 with the Lavaan package version 05.14. Run this file.
        i. This will create a new file: 'STAP_latent.txt' containing the latent scores for all (new) participants.
2. The file 'STAP_latent.txt' should be saved as excel file before exporting in SPSS. moet Open this file in excel and save it as excel work folder. Column names may have moved incorrectly, and should all be moved one column to the right. The first colum (without name) can be deleted. Save this file as 'STAP_latent.xls'
3. Import 'STAP_latent.xls' in SPSS.
4. The file 'eyetracker_noise_split.sav' contains split-half variables and noise variables on the eye tracker data. This file can be merged with the SPSS file made from 'STAP_latent.xls', and saved as 'eyetracker_totaal.sav'.

---

[1] De Jong M, Verhoeven M, Hooge, ITC, Van Baar AL. Introduction of the Utrecht Task for Attention in Toddlers Using Eye Tracking (UTATE): A Pilot Study. *Front Psychol.* 2016a;7:669. doi: 10.3389/fpsyg.2016.00669

[2] Nyström, M., & Holmqvist, K. (2010). An adaptive algorithm for fixation, saccade, and glissade detection in eyetracking data. *Behavior Research Methods, 42,* 188-204. doi:10.3758/BRM.42.1.188

[3] Smeets, J.B.J., & Hooge, I.T.C. (2003). Nature of variability in saccades. *Journal of Neurophysiology, 90,* 12-20. doi:10.1152/jn.01075.2002

[4] Hooge I, Camps G. Scan path entropy and arrow plots: Capturing scanning behavior of multiple observers. *Front Psychol.* 2013;4:996.doi:10.3389/fpsyg.2013.00996

[5] De Jong M, Verhoeven M, Hooge ITC, Van Baar AL. Factor Structure of Attention Capacities. J Atten Disord. 2016b;20(3):230– 239. doi:10.1177/1087054713516002.

[6] Van De Schoot R, Lugtig P, Hox J (2012). A checklist for testing measurement invariance. Eur J Dev Psychol. 2012:9, ¬486-492.
